# Aflatoxin Reduction and Retardation of Aflatoxin Production by Microorganisms in *Doenjang* during a One-Year Fermentation

**DOI:** 10.3390/jof8020190

**Published:** 2022-02-15

**Authors:** Vishal Kumar, Ashutosh Bahuguna, Srinivasan Ramalingam, Jong Suk Lee, Sung Soo Han, Hyang Sook Chun, Myunghee Kim

**Affiliations:** 1 Department of Food Science and Technology, Yeungnam University, Gyeongsan 38541, Gyeongsangbuk-do, Korea; vkaggarwal180@gmail.com (V.K.); ashubahuguna@gmail.com (A.B.); sribt27@gmail.com (S.R.); 2 Division of Food & Nutrition and Cook, Taegu Science University, Daegu 41453, Gyeongsangbuk-do, Korea; jslee1213@ynu.ac.kr; 3 School of Chemical Engineering, Yeungnam University, Gyeongsan 38541, Gyeongsangbuk-do, Korea; sshan@yu.ac.kr; 4 Institute of Cell Culture, Yeungnam University, Gyeongsan 38541, Gyeongsangbuk-do, Korea; 5 School of Food Science and Technology, Chung-Ang University, Anseong 17546, Gyeonggi-do, Korea; hschun@cau.ac.kr

**Keywords:** *doenjang*, fermentation, aflatoxin, *Aspergillus flavus*, degradation, fungi, bacteria

## Abstract

*Meju,* a raw material for *doenjang* preparation, is highly vulnerable to aflatoxin-producing fungi. The aim of this study was to evaluate the effect of a one-year fermentation on aflatoxins and aflatoxin-producing fungi in *doenjang* spiked with aflatoxins B1, G1, B2, and G2 and inoculated with toxigenic *Aspergillus flavus*. A significant reduction in aflatoxins was observed after a year of fermentation, measuring 92.58%, 100%, 98.69%, and 100% of B1, G1, B2, and G2, respectively. After a year of fermentation, 6.95 ± 3.64 µg/kg of total aflatoxin was detected, which represents a 97.88% reduction in the total aflatoxin compared with the initial value (328.83 ± 36.60 µg/kg). Several aflatoxin-degrading fungi (*Aspergillus versicolor*, *Cladosporium subcinereum*, *Aspergillus ochraceus*) and bacteria (*Bacillus albus*, *Bacillus velezensis*) isolated from *doenjang* were identified as the major contributors to the reduction of aflatoxin. Furthermore, it was observed that most of the aflatoxin contamination in *doenjang* occurred during the *meju* stage, and this stage was found to be most susceptible to *A. flavus* contamination and growth. These findings reveal that native microorganisms mediate aflatoxin clean-up in *doenjang* during fermentation and support the use of such microorganisms as a starter culture for the preparation of aflatoxin-free *doenjang*.

## 1. Introduction

Soybean-based fermented foods are an integral part of the diet in many Southeast Asian countries, including Korea, China, and Japan [1,2,3]. Examples of soybean-based fermented foods include *thua nao* from Thailand, *sufu* from China, *natto* from Japan, *tempeh* from Indonesia, and *doenjang* from Korea. *Doenjang* is a traditional fermented soybean paste prepared using *meju* as a starter culture. *Doenjang* quality is influenced by its raw ingredients and the fermenting microbial communities [4,5]. *Doenjang* is made of *meju* and salt and is naturally fermented by diverse microbial communities. Both aerobic and anaerobic fermentation take place during *doenjang*-*meju* preparation. In the exterior part of *doenjang-meju*, aerobic fermentation takes place, whereas in the interior part, anaerobic or microaerophilic fermentation is predominant. Microbial communities also vary greatly in the interior and exterior parts of *doenjang*-*meju* fermentation. The outer part is rich in aerobic microorganisms such as *Bacillus*, whereas the internal part is abundant in anaerobic, microaerobic, and facultative anaerobic bacteria such as *Enterococcus*, *Lactobacillus*, *Clostridium*, and *Myroides* [6]. Occasionally, *meju* can be contaminated with aflatoxin-producing fungi such as *Aspergillus flavus* and *Aspergillus parasiticus*, leading to the contamination of *doenjang* with aflatoxins [7]. *Aspergillus flavus* produces aflatoxins B1 and B2, whereas *A. parasiticus* produces aflatoxins G1 and G2 [8,9]. Aflatoxin contamination poses a serious challenge to food safety, as it is associated with several health hazards. Aflatoxin B1 exerts strong mutagenicity and carcinogenicity; it is classified as a Group 1 human carcinogen by the International Agency for Research on Cancer [10,11]. The Korea Food and Drug Administration limits total aflatoxin and aflatoxin B1 levels in *doenjang* to 15 µg/kg and 10 µg/kg, respectively, to ensure safe consumption.

*Doenjang* is commonly consumed in Korea and is prepared either commercially or traditionally at home. Homemade *doenjang* is often prepared using traditional fermentation methods involving natural microflora. However, commercial *doenjang* is prepared in a controlled environment using selected microbial strains under strict fermentation conditions [12]. Thus, homemade *doenjang* is more vulnerable to contamination by undesired microorganisms. Therefore, it is necessary to understand the changes in aflatoxin levels that occur during the fermentation of traditional *doenjang* to ensure the safety of *doenjang* consumption. This study was conducted to examine aflatoxin levels in *doenjang* prepared by fermentation over a year. Furthermore, *doenjang* was artificially contaminated with aflatoxins and inoculated with aflatoxin-producing fungi (*A. flavus*) to examine the effect of fermentation on aflatoxin levels, fungal growth, and aflatoxin production. Additionally, different microorganisms (bacteria and fungi) were isolated from *doenjang* and examined for their aflatoxin degradation ability to develop an effective starter culture for the preparation of aflatoxin-free *doenjang*.

## 2. Materials and Methods

### 2.1. Materials

Salt and soybean used for *doenjang* preparation were purchased from a local market (Gyeongsan, Gyeongsangbuk-do, Korea). Immunoaffinity columns for aflatoxin purification were supplied by VICAM (Milford, MA, USA). Acetonitrile and methanol for high-performance liquid chromatography (HPLC) were supplied by Sigma-Aldrich (St. Louis, MO, USA). Whatman filters (0.22 µm) were used to filter HPLC solvents. The standard aflatoxins B1, G1, B2, and G2 were purchased from LIBIOS (Vindry-sur-Turdine, France). The aflatoxin-producing strain *A. flavus* KACC45470 was procured from the Korean Agricultural Culture Collection (KACC, Suwon, Korea). Dulbecco’s Modified Eagle’s Medium (DMEM) and fetal bovine serum (FBS) were procured from Gibco (Waltham, MA, USA). 3-(4,5-Dimethylthiazol-2-yl)-2,5-diphenyltetrazolium bromide (MTT) and antibiotics were purchased from Invitrogen (Waltham, MA, USA). Dimethyl sulfoxide (DMSO) was purchased from Sigma-Aldrich (St. Louis, MO, USA). Human skin fibroblast cells were procured from the American Type Culture Collection (ATCC, Manassas, VA, USA).

### 2.2. Preparation of Doenjang

**Figure 1:**
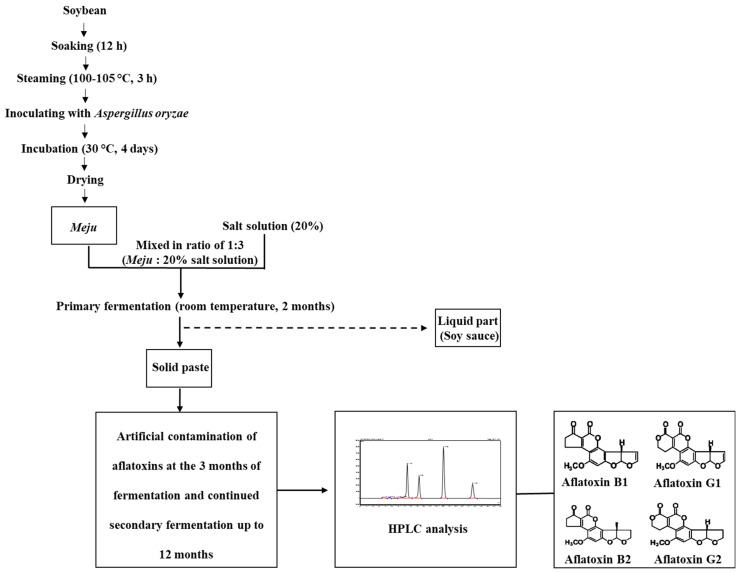
Flow diagram representing the experimental design for *doenjang* preparation, aflatoxin spiking, and aflatoxin analysis in *doenjang*.

*Meju* was prepared as depicted in Figure 1. Soybeans were soaked in water for 12 h and then steamed at 100–105 °C for 3 h. The steamed soybeans were cooled at room temperature (25.0 ± 2.5 °C), mixed with an *Aspergillus oryzae* culture, and incubated for 4 days at 30 °C, followed by drying. The prepared *meju* was mixed with three-fold of 20% salt solution and fermented at room temperature for 2 months. After primary fermentation, the liquid part (soy sauce) was separated, and the solid soybean paste (*doenjang*) was further fermented for 12 months at room temperature, during which samples were collected for aflatoxin analysis.

### 2.3. Artificial Contamination of Doenjang

To evaluate the effect of the *doenjang* matrix on aflatoxin reduction, artificial contamination experiments were carried out as follows.

#### 2.3.1. Artificial Contamination of Commercial Aflatoxins

*Doenjang* was artificially contaminated with aflatoxins B1, G1, B2, and G2. Each aflatoxin was prepared at a concentration of 60 µg/mL; 500 µL of the aflatoxin mixture was added to 300 g of *doenjang* samples to obtain a final concentration of 100 µg/kg for each aflatoxin. After mixing, all samples were incubated at room temperature for 12 months. Experiments were carried out in three independent experiments, each containing 300 g of *doenjang* spiked with 100 µg/kg of each aflatoxin. Before the sample collection, aflatoxin-spiked *doenjang* was mixed thoroughly, and 15 g of the sample was collected from each of the triplicates. The remaining samples were kept for further fermentation and collection of the samples at different fermentation times. Additionally, a control containing aflatoxin-spiked *doenjang* was maintained at 4 °C to stop the activity of microorganisms and fermentation. Furthermore, *doenjang* was autoclaved to eradicate the native microorganisms and then spiked with aflatoxins. The aflatoxin-spiked autoclaved *doenjang* was stored at room temperature and served as the control. Aflatoxin levels in the control samples were analyzed after 12 months.

#### 2.3.2. Artificial Contamination of *A. flavus*

A toxigenic strain of *A. flavus* was grown at 28 °C for seven days in potato dextrose broth (PDB) in a shaking incubator (150 rpm) to obtain the mycelial biomass. The mycelial biomass was separated through filtration using a double-layered muslin cloth and washed twice with double-distilled water. Finally, mycelial biomass (7.5 g) was added to 300 g of *doenjang* samples. The experiments were performed in three independent experiments. Before the sample collection, *A. flavus-*inoculated *doenjang* was mixed thoroughly, and 15 g of the sample was collected from each of the triplicates. The remaining samples were kept for further fermentation and collection at different fermentation times.

### 2.4. Aflatoxin Extraction and HPLC Analysis

*Doenjang* samples (10 g) were homogenized for 2 min at 10,000 rpm in 50 mL of 80% methanol. This was followed by the addition of 25 mL hexane and 30 min of shaking at room temperature. The sample was filtered through Whatman filter paper (No. 4), and the filtrate was further diluted by eight-fold using triple-distilled water containing 0.1% Tween 20. The diluted filtrate was passed through a glass fiber filter (1.6 µm) to obtain a clear solution. Then, 20 mL of the filtered solution was passed through the VICAM AflaTest affinity column to bind the aflatoxins. The affinity column was then washed with 10 mL of distilled water to remove impurities and salts. After 30 min, the aflatoxins were eluted using 4 mL of methanol containing 0.1% acetic acid. The eluents were dried using N_2_ gas, reconstituted in 600 µL of 20% acetonitrile:trifluoroacetic acid (4:1) solution, and filtered using a 0.2 µm syringe filter. Aflatoxins were quantified using an UltiMate 3000 HPLC system (Thermo Fisher Scientific, Waltham, MA, USA). A Cloversil-C18 column (4.6 mm width × 250 mm length; pore size 5 μm; Shiseido Co., Ltd., Tokyo, Japan) was used for separation at a column temperature of 30 °C. The injection volume was 20 μL, and the mobile phase comprised water:acetonitrile:methanol (3:1:1, *v*/*v*). The flow rate was maintained at 0.8 mL/min. The excitation and emission wavelengths of the spectra were 360 nm and 450 nm, respectively. For the quantification of aflatoxin in *doenjnag* samples, a calibration curve was prepared using the standard aflatoxins B1, G1, B2, and G2 (Figure S1 and Table S1).

### 2.5. Aflatoxin Production by A. flavus at Different Stages of Doenjang Preparation

*A. flavus* spores were inoculated at three different stages of *doenjang* preparation: *meju* formation and primary and secondary fermentation stages (Figure 1). For *meju* preparation, ~10^6^ spores of *A. flavus* were inoculated in 200 g of boiled and crushed soybeans. *Meju* bricks were prepared and stored at room temperature. Then, *meju* was examined for aflatoxin production at different time points (0 day, 10 days, 20 days, and 40 days). For the *A. flavus* inoculation at the primary fermentation stage, 200 g of commercial *meju* was inoculated with the spores of *A. flavus* (~10^6^), and finally, the *meju* was dipped in the 20% salt solution and stored at room temperature. This *meju* was analyzed for aflatoxin production at 0 day, 15 days, 30 days, 45 days, and 60 days of fermentation. For the secondary fermentation, 60 days-aged primary fermented *meju* (200 g) was inoculated with *A. flavus* (~10^6^) and incubated at room temperature for 6 months. Fermented samples were collected and processed for aflatoxin quantification after 1 month, 2 months, 3 months, 4 months, 5 months, and 6 months.

### 2.6. Aflatoxin Degradation by Microflora from Doenjang

The native microorganisms present in *doenjang* were extracted by suspending 1 g of *doenjang* sample in 9 mL of 0.85% saline. After 2 h of shaking, 0.5% (*v/v*) of the extracted suspension was inoculated in nutrient broth spiked with 100 ng/mL each of aflatoxins B1, G1, B2, and G2. After 5 d of incubation at 30 °C, the culture supernatant and pellet fractions were collected individually by centrifugation at 10,000 rpm for 10 min. Both fractions were analyzed separately to detect the residual aflatoxins. *Doenjang* samples showing aflatoxin degradation were further processed for the isolation of microorganisms (bacteria and fungi) using the serial dilution method. Briefly, 1 g of *doenjang* was mixed with 9 mL of 0.85% saline and serially diluted to a 10^−7^ dilution. Samples from each dilution were spread on nutrient agar plates and incubated at 30 °C for 48 h to allow the growth of bacteria. Similarly, samples from each dilution were spread onto a potato dextrose agar plate containing kanamycin (50 µg/mL) and incubated at 28 °C for 7 days to allow the growth of fungi. Different bacterial and fungal strains were isolated by repetitive streaking to obtain a pure culture.

All bacterial isolates were cultured for 48 h in nutrient broth at 30 °C in a shaking incubator at 150 rpm. Cell-free supernatant (CFS) was collected by centrifuging the culture broth at 10,000 rpm for 10 min. The CFS of bacterial isolates was then spiked with the aflatoxin mixture (B1, G1, B2, and G2 at a concentration of 1000 ng/mL each) and incubated for 7 days at 35 °C. After seven days of incubation, aflatoxins were extracted from the aflatoxin-mixture-spiked CFS and analyzed using HPLC, as described in Section 2.4.

All fungal isolates were cultured for 15 days in potato dextrose broth at 28 °C in a shaking incubator at 150 rpm. CFS was collected by centrifuging the culture broth at 10,000 rpm for 10 min. The CFS of fungal isolates was then spiked with the aflatoxin mixture (B1, G1, B2, and G2 at a concentration of 1000 ng/mL each) and incubated for 7 days at 35 °C. After seven days of incubation, aflatoxins were extracted from the aflatoxin-mixture-spiked CFS and analyzed using HPLC, as described in Section 2.4.

### 2.7. Detection of Aflatoxin Production by Fungi Isolated from Doenjang

All the fungal isolates from *doenjang* were analyzed for the aflatoxin (B1, G1, B2, G2) production by inoculating the mycelium directly into 50 mL PDB, followed by 7 d of incubation at 28 °C in a rotatory shaker (150 rpm). Finally, the content was quantified for the production of aflatoxins (B1, G1, B2, G2) by using HPLC, as per the method adopted in Section 2.4.

### 2.8. Effect of Salt Concentration on Aflatoxin-Producing Fungi

The aflatoxin-producing fungal isolates from *doenjang* and standard aflatoxin-producing strains of *A. flavus* were tested for their salt tolerance capabilities. The fungal spores of the respective isolates were inoculated in PDB containing different salt concentrations (12%, 14%, 16%, 18%, and 20%). After 15 days incubation at 28 °C, growth and mycotoxin production were analyzed at each salt concentration.

### 2.9. Molecular Identification of Microorganisms Isolated from Doenjang

Bacteria and fungi isolated from *doenjang* were identified via 16S rRNA gene and internal transcribed spacer sequencing, respectively, at the SolGent (Daejeon, Korea) commercial sequence facility. The amplified 16S rRNA genes and internal transcribed spacer sequences of the isolated bacteria and fungi, respectively, were searched for similarities in the NCBI GenBank database using the nBLAST tool. Sequences showing high similarity with the query sequences were aligned in CLUSTALW using the Mega6.0 software [13] and processed for phylogenetic analysis using the neighbor-joining method.

### 2.10. Toxicity Analysis of Aflatoxin and Aflatoxin Degradation Products in Doenjang 

The toxicity of the aflatoxin and aflatoxin degradation products in *doenjang* was determined by examining human skin fibroblast cell viability.

#### 2.10.1. Cells and Cell Culture

Human skin fibroblast cells were maintained in DMEM containing 10% (*v*/*v*) FBS and a cocktail of penicillin and streptomycin. The cells were maintained at 37 °C in the presence of 5% CO_2_.

#### 2.10.2. Extraction of Aflatoxin and Its Degradation Products form *Doenjang*

Five grams of *doenjang* (samples were collected at zero days and twelve months fermented) was homogenized with ten milliliters of distilled water, followed by mixing with twenty milliliters of hexane. The *doenjang* suspension was shaken at 120 rpm in a shaking incubator. After 30 min, the hexane phase was removed, and the aqueous suspension was mixed with 15 mL of chloroform to extract the aflatoxin and aflatoxin degradation products. Finally, chloroform was evaporated at 50 °C under a gentle stream of N_2_ gas, and the dried samples were dissolved in 100 µL of DMSO.

#### 2.10.3. Toxicity Determination using Human Skin Fibroblast Cell Viability

The toxicity of the aflatoxin and aflatoxin degradation products extracted from *doenjang* was examined by the MTT assay [14]. Briefly, human skin fibroblast cells (1 × 10^4^ cells/mL) were seeded in a 96-well culture plate and incubated for 24 h at 37 °C in a CO_2_ incubator. The cells were subsequently treated with 15 µL of samples extracted from *doenjang* on Day 0 and 12 months fermented products. After 24 h of incubation, cells were mixed with 10 µL of MTT solution (5 mg/mL) for 4 h. As a result, the formazan crystals formed and were dissolved in DMSO, and the cell viability was determined by measuring the absorbance at 540 nm. The results are represented as the percentage of viable cells. Human skin fibroblast cells without any treatment were used as the control, and their cell viability was considered 100%.

### 2.11. Statistical Analyses

All experiments were performed in triplicate, and the results are presented as the mean ± standard deviation. The SPSS-16 software (IBM, Chicago, IL, USA) was used to evaluate the statistical significance between groups, employing ANOVA and the Duncan test for post hoc analysis at *p* < 0.05.

## 3. Results and Discussion 

### 3.1. Aflatoxin Analysis in Non-Contaminated Doenjang during Fermentation

Fermented foods such as *doenjang* are vulnerable to mycotoxin contamination because of the long duration of fermentation in the natural environment [15]. Mycotoxins, such as aflatoxins, are the most serious toxicants in fermented foods because of their potential toxicity to humans and animals [9,16,17]. Several studies have reported the presence of aflatoxins in different *doenjang* samples [15,18]. Therefore, it is imperative to examine the presence of such toxins to determine the safety of fermented food consumption. In the present study, we examined the presence of aflatoxins B1, G1, B2, and G2 in *doenjang* samples during 12 months of fermentation. The *doenjang* preparation was almost free from aflatoxin contamination. However, a non-considerable (below the limit of quantification) (Table S1) amount of aflatoxins was traced in a few samples (Table S2), which spontaneously disappeared with the fermentation time. The disappearance of aflatoxins during the fermentation could be due to the presence of different microorganisms that have the potential to degrade aflatoxins, or they could be antagonistic towards mycotoxin-producing fungi. In addition, several physical and chemical factors that arise during fermentation may have contributed to the effective reduction of aflatoxins [19,20].

### 3.2. Aflatoxin Analysis in Artificially Contaminated Doenjang during Fermentation

To examine the effect of the microflora, fermentation time, and *doenjang* matrix on aflatoxin reduction, *doenjang* samples were artificially contaminated with commercial aflatoxins B1, G1, B2, and G2. In addition, *doenjang* samples were inoculated with aflatoxin-producing *A. flavus* to examine their mycotoxin-producing behavior during the fermentation. Three-month-old *doenjang* samples were used to carry out this experiment, as the samples at this stage contained trace amounts of mycotoxins, which disappeared eventually, thus providing a preliminary indication that they may contain some microorganisms that could effectively reduce the mycotoxins. All aflatoxin-spiked *doenjang* samples were fermented for 12 months at room temperature to monitor the changes in aflatoxin levels.

**Figure 2:**
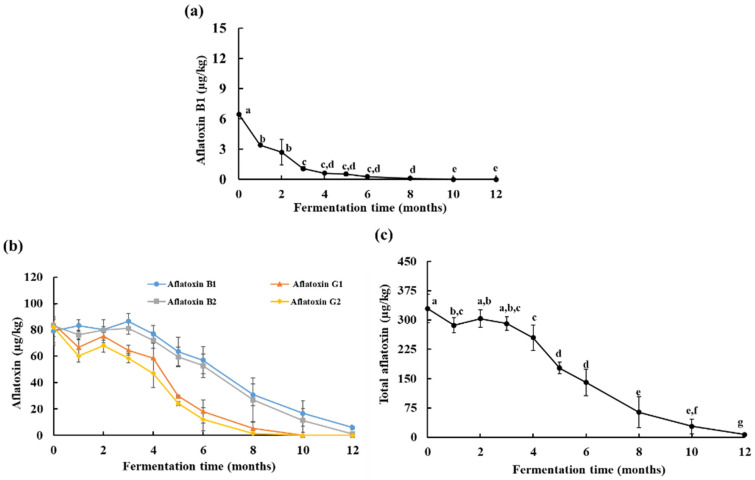
Analysis of aflatoxin in *Aspergillus flavus*-inoculated and aflatoxin-spiked (100 µg/kg each of aflatoxins B1, G1, B2, and G2) *doenjang* samples during 12 months of fermentation. (**a**) Quantification of aflatoxin B1 in *Aspergillus flavus* inoculated *doenjang* during 12 months of fermentation. (**b**) Time-dependent quantification of aflatoxins B1, G1, B2, and G2 in aflatoxin-spiked *doenjang* samples. (**c**) Time-dependent quantification of total aflatoxin in aflatoxin-spiked *doenjang* samples. Each value represents the mean ± standard deviation of three independent experiments. Different letters (a–g) represent a significant difference at *p* < 0.05.

On the initial day, the control *doenjang* sample (not spiked with mycotoxins) showed a trace amount (<0.50 µg/kg) of aflatoxin, which eventually disappeared after 1 month of fermentation. On the initial day, commercial aflatoxin-spiked *doenjang* was recovered with 79.07 ± 10.55 µg/kg aflatoxin B1, 84.54 ± 14.40 µg/kg aflatoxin G1, 83.29 ± 4.56 µg/kg aflatoxin B2, and 81.92 ± 7.09 µg/kg aflatoxin G2 (Figure 2b). After 12 months, aflatoxins B1 and B2 were reduced to 5.86 ± 1.84 µg/kg and 1.09 ±1.80 µg/kg, respectively, in *doenjang*. A complete reduction of aflatoxins G1 and G2 was observed after 12 months of fermentation (Figure 2b). During 12 months, the total aflatoxin levels were reduced to 6.95 ± 3.64 µg/kg from the 328.83 ± 36.6 µg/kg detected at the beginning of fermentation (Figure 2c). These findings suggest a periodic reduction of aflatoxins during fermentation, which might be due to the presence of diverse microflora in *doenjang* that can degrade aflatoxins. Previously, the presence of aflatoxin-degrading microorganisms had been documented in various fermented foods [21,22], which supports our assumption that diverse microflora present in *doenjang* are the major contributors to the reduction of aflatoxins. Nevertheless, no study to date has demonstrated the involvement of microorganisms in the reduction of aflatoxins in *doenjang*. A detailed study confirming microorganism-mediated aflatoxin degradation is carried out in a later section.

In the *A. flavus* inoculated samples, 6.45 ± 0.05 µg/kg of aflatoxin B1 was detected on the first day, which was completely disappeared after 12 months of fermentation (Figure 2a). These results were surprising, as aflatoxin production was expected during the fermentation because *doenjang* samples were inoculated with aflatoxin-producing *A. flavus*. These results might be due to the high salt concentration of *doenjang,* which restricts the growth of *A. flavus* and, consequently, the production of aflatoxin. In addition, it might be possible that *doenjang* contains microorganisms that have an inhibitory effect on *A. flavus*. This is consistent with earlier reports demonstrating the *A. flavus*-inhibitory role of the microorganisms present in *doenjang* [23,24,25]. The findings indicated a non-conducive environment for *A. flavus* growth and aflatoxin production in *doenjnag*, and as a result, no production of aflatoxin was detected. Moreover, the *A. flavus* growth and aflatoxin production were also affected by the aerobic and anaerobic fermentation conditions [26]. Generally, the growth of *A. flavus* and aflatoxin production are negatively correlated with CO_2_ concentration. In a study, it was observed that *A. flavus* growth was effectively reduced at a 25% CO_2_ level and aflatoxin production was significantly reduced at a 50% CO_2_ level in high-moisture maize grains [27]. Similarly, a 59% and an 88% reduction in *A. flavus* growth and a 47% and a 97% reduction in aflatoxin production in paddy by a 20% and an 80% CO_2_ level were observed, respectively [28]. However, in the present study, *doenjnag* was kept in boxes where gases could pass freely, therefore ruling out the possibilities of CO_2_ accumulation and its negative impact on *A. flavus* growth and production of aflatoxin. Additionally, even on the surface, where plenty of oxygen was available, *A. flavus* growth and aflatoxin production were not observed, which confirmed that CO_2_ or O_2_ was not the limiting factor in the present study. Furthermore, *A. flavus* was not able to grow or produce aflatoxin at a high salt concentration (>12%) in PDB medium where enough oxygen was available, also supporting the current findings. Therefore, these observations ruled out the possibility of the inhibition of *A. flavus* growth and aflatoxin production due to anaerobic conditions. 

### 3.3. Aflatoxin Production by A. flavus at Different Stages of Doenjang Preparation

**Figure 3:**
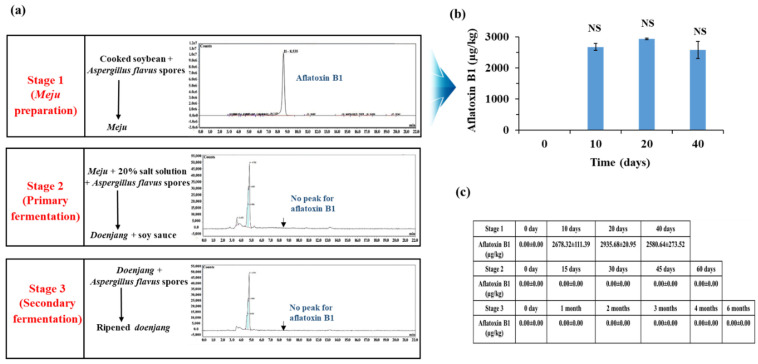
Aflatoxin production by *Aspergillus flavus* at three different stages of the *doenjang* preparation. (**a**) Aflatoxin production at the *meju* stage (Stage 1), primary fermentation (Stage 2), and secondary fermentation (Stage 3). (**b**) Time-dependent aflatoxin B1 production at the *meju* stage. Values in the bar graph represent the mean ± standard deviation of three independent experiments. (**c**) The combined results representing the time-dependent aflatoxin production by *Aspergillus flavus* at three different stages of *doenjang* preparation. NS: non-significant difference at *p* < 0.05.

Aflatoxins were not detected in *A. flavus-*contaminated *doenjang,* indicating that *A. flavus* is not growing or is not producing aflatoxin in the *doenjang* environment. To evaluate and identify the stage of *doenjang* preparation that is most vulnerable to the *A. flavus* contamination and aflatoxin production, *A. flavus* was artificially inoculated at three different stages of *doenjang* preparation such as *meju* preparation, primary fermentation, and secondary fermentation stages. The analysis of the samples from different *doenjang* fermentation stages revealed that the *meju* preparation step was the most favorable stage for the growth of *A. flavus* and the production of aflatoxin. In the *meju* sample, an intense growth of *A. flavus* was detected (macroscopically). In contrast to this, no visible growth of *A. flavus* was detected in primary and secondary fermented samples. The *meju* samples inoculated with *A. flavus* showed 2678.32 ± 111.39 µg/kg of aflatoxin B1 after 10 days of incubation (Figure 3b). In contrast, no aflatoxin production was detected in primary and secondary fermentation samples (Figure 3a,c). This suggests that the primary and secondary fermentation stages of the *doenjang* preparation do not favor the growth of *A. flavus* owing to the high salt concentrations. A similar study was carried out to observe *A. flavus* growth and aflatoxin production in peanuts, and it reported that 4% NaCl inhibited aflatoxin production by *A. flavus* [29], which supports the current findings.

### 3.4. Detection of Aflatoxins in Fungi Isolated from Doenjang

Most of the *doenjang* samples inoculated with aflatoxin-producing *A. flavus* showed little or no presence of mycotoxins (Figure 2a), suggesting that the *doenjang* environment is unfavorable for their growth and/or aflatoxin production. In the control group, most of the samples were detected for trace amounts of aflatoxin, suggesting the presence of certain native aflatoxin-producing fungi. Therefore, different fungi were isolated from the *doenjang* samples and examined for aflatoxin production. Based on the colony morphology and culture characteristics, 14 different fungi were detected in different *doenjang* samples, which were further analyzed for aflatoxin production. Among the fourteen isolated strains, only three isolates (YURM3, YURM4, and YURM9) produced aflatoxins B1 and B2. Isolates YURM3, YURM4, and YURM9 produced 41.19 µg/L, 76.61 µg/L, and 109.94 µg/L of aflatoxin B1 and 1.06 µg/L, 0.95 µg/L, and 0.10 µg/L of aflatoxin B2. The remaining isolates did not produce any aflatoxin. The three aflatoxin-producing fungi were examined microscopically for tentative identification. Microscopic and culture characteristic examination revealed identical features of YURM3 and YURM9, which resembled the classical features of *A. flavus.* Therefore, both isolates were suspected to be *A. flavus.* However, the characteristics of YURM4 matched with the typical features of *Aspergillus ruber;* hence, it was tentatively identified as *A. ruber.* Further, the identification of these aflatoxin-producing strains was confirmed by molecular characterization based on internal transcribed spacer sequencing (Table 1).

### 3.5. Effect of Salt on the Growth of Aflatoxin-Producing Fungi

As *doenjang* contains a high amount of salt (~20%) [30], the growth and aflatoxin-producing efficiency of YURM3, YURM4, and YURM9 and the standard aflatoxin-producing strain *A. flavus* were analyzed at different salt concentrations. The results revealed that the growth of YURM3, YURM4, and YURM9 beyond a 12% salt concentration was not observed, suggesting that salt concentrations higher than 12% are an effective barrier to prevent the growth of these toxigenic isolates. Consistent with the growth, aflatoxin production was observed only up to a salt concentration of 12%. Similarly, the medium without salt showed higher growth and aflatoxin production than the medium containing 12% salt (Table 1). Similar results were observed with the standard *A. flavus* strain, where growth and aflatoxin production were observed only up to a 12% salt concentration. These results suggest that the high amount of salt in *doenjang* prevents the growth of these toxigenic fungi, resulting in low or no aflatoxin production.

### 3.6. Confirmation of Aflatoxin Degradation in Doenjang

**Figure 4:**
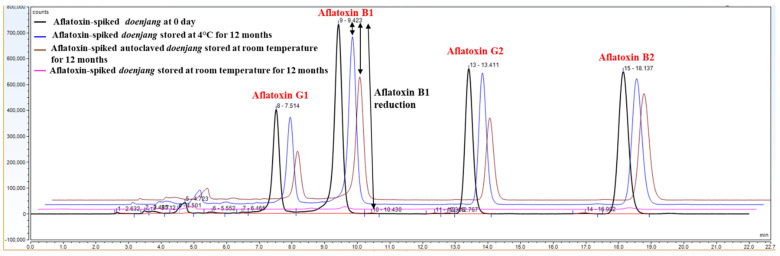
HPLC chromatograms representing aflatoxin levels in *doenjang* spiked with 100 µg/kg each of aflatoxins B1, G1, B2, and G2 on Day 0 and after 12 months of storage at 4 °C and room temperature (25 ± 2.5 °C).

The control *doenjang* samples were stored in a refrigerator to prevent microbial activity. Additionally, a set of *doenjang* samples was autoclaved to eradicate the existing microorganisms and stored at room temperature. After 12 months, the stored samples were analyzed for the presence of residual aflatoxins (Figure 4). As a result, 75.18 ± 0.73 µg/kg of aflatoxin B1, 71.65 ± 3.24 µg/kg of aflatoxin G1, 74.15 ± 0.11 µg/kg of aflatoxin B2, and 74.67 ± 4.26 µg/kg of aflatoxin G2 were recovered from the refrigerated *doenjang* samples (Figure S2a). The recovered amounts of aflatoxins were nearly the same as those recovered from *doenjang* samples collected on day 0, indicating that there was no degradation during the storage at 4 °C (Figure S2b). From the autoclaved *doenjang* samples, 53.58 ± 0.36 µg/kg of aflatoxin B1, 40.68 ± 0.77 µg/kg of aflatoxin G1, 65.03 ± 0.17 µg/kg of aflatoxin B2, and 45.26 ± 0.95 µg/kg of aflatoxin G2 were recovered after 12 months of storage at room temperature (Figure S2a). The total aflatoxin recovered from the refrigerator-stored *doenjang* and the autoclaved *doenjang* stored at room temperature was 42- and 30-times higher, respectively, than that of the un-autoclaved *doenjang* stored at room temperature for 12 months (Figure S2b). The comparative result obtained from refrigerator-stored, room temperature-stored, autoclaved, and un-autoclaved *doenjang* samples implied a key involvement of native microorganisms in reducing aflatoxins during storage. As most of the microorganisms failed to grow at 4 °C, no degradation of aflatoxins was noticed in refrigerator-stored *doenjang.*

The autoclaved *doenjang* had a microorganism-free environment, but still showed some degree of aflatoxin degradation, suggesting the contribution of factors such as the *doenjang* matrix and physicochemical properties to degrade aflatoxin besides the sole involvement of microorganisms. The influence of microorganism’s activity in both 4 °C and autoclaved *doenjang* was suppressed; however, the physical factors such as temperature contributed significantly to the degradation of aflatoxin, evident from the detected aflatoxin from un-autoclaved *doenjang* stored at room temperature. In autoclaved *doenjang*, temperature (25 ± 2.5 °C) played a critical role to stimulate the physiochemical-induced aflatoxin degradation. The recovered aflatoxin from autoclaved and un-autoclaved *doenjang* stored at 12 months suggested that the majority of aflatoxin degradation was stimulated by microorganisms, though the physiochemical conditions also contributed significantly. Among the different chemical factors, the salt content of *doenjang* may be responsible for aflatoxin degradation, and this notion was supported by the in vitro study, where a significant amount of aflatoxin was degraded in the presence of a 20% salt solution during the storage of 60 days at room temperature (data not shown). These results collectively suggest that the microbial population is responsible for reducing the aflatoxin levels during fermentation. However, the physicochemical properties of *doenjang* also contribute to the degradation of aflatoxin to some degree.

These results are consistent with that demonstrated aflatoxin degradation in soy-based products during fermentation primarily by microorganisms [31,32]. Similarly, Jeong et al. (2019) demonstrated aflatoxin reduction in *doenjang* during fermentation [9]. Lee et al. (2017) also observed aflatoxin reduction in *doenjang* and suggested the possibility of certain aflatoxin detoxifying microorganisms during fermentation [24]. Lee et al. (2017) isolated *A. oryzae* MAO 103 and MAO 104 from *doenjang*, which were able to degrade aflatoxins [24]. Consistent with this, Petchkongkaew et al. (2008) isolated 23 *Bacillus* species from soybean and fresh Thua-nao collected from the north of Thailand and observed that most of the strains could detoxify aflatoxin [33]. These results also support the present findings that fermented soybean-based products contain aflatoxin-degrading microorganisms. The results of the current findings indicate microorganisms as the major contributor to reducing aflatoxin in *doenjang*. However, other factors involved in aflatoxin degradation need to be explored in detail to decode the aflatoxin degradation mechanism.

### 3.7. Aflatoxin Degradation by the Microbial Consortium Obtained from Doenjang

**Figure 5:**
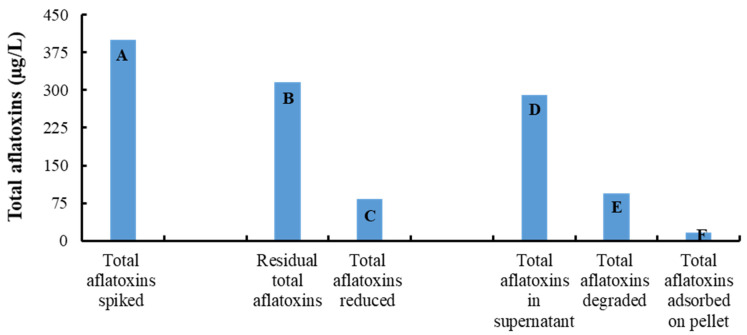
Degradation of aflatoxin (100 µg/kg each of aflatoxins B1, G1, B2, and G2) by the microbial consortium obtained from *doenjang*. The bar column (A) represents the amount of total aflatoxins spiked. The bar columns (B) and (C) represent total residual aflatoxin (B) and the corresponding amount of the reduced aflatoxin (C), respectively, after 5 days of incubation. The bar columns (D), (E), and (F) represent total aflatoxin in the cell-free supernatant (D), degraded aflatoxin (E), and aflatoxin adsorbed by the microbial consortium obtained from *doenjang* (F), respectively.

The whole native microbial population was obtained from *doenjang* applied for aflatoxin degradation in nutrient broth. After five days of incubation, the residual aflatoxins were detected in the culture broth, CFS, and bacterial cell pellets. There was 316.44 µg/L of total residual aflatoxins detected in the nutrient broth inoculated with extracted microorganisms against the initially spiked 400 µg/L of the total aflatoxins, representing a 20.89% reduction in the total aflatoxin levels (Figure 5). Specifically, 90.03 µg/L of residual aflatoxin B1 and 92.42 µg/L of residual aflatoxin B2 were detected in the whole culture broth of *doenjang*, which was initially spiked with 100 µg/L of each aflatoxin. In contrast, much smaller amounts of residual aflatoxin G1 (64.15 µg/L) and residual aflatoxin G2 (69.84 µg/L) were observed. These results indicate that aflatoxins G1 and G2 were more vulnerable to microbial degradation compared to aflatoxins B1 and B2. Furthermore, a considerable amount of total aflatoxins (15.59 µg/L) was detected in the bacterial pellet, suggesting that certain microbes present in the *doenjang* samples can adsorb aflatoxins as well. As shown in Figure 5, the adsorbed amount of total aflatoxin was very low, suggesting that microbial degradation is the major cause of the reduction in aflatoxin levels in *doenjang* samples. *Doenjang* is rich in diverse microflora such as aerobic bacteria, *Lactobacillus*, yeast, and fungi. Aerobic bacteria generally belong to the *Bacillus* group, the members of which are known to degrade aflatoxins [33,34]. The results correlated with our earlier findings demonstrating a time-dependent reduction of aflatoxins in *doenjang* samples and strongly support the notion that microorganisms in *doenjang* are the key contributors to reducing the aflatoxin levels.

### 3.8. Degradation of Aflatoxin by Microbial Isolates

**Figure 6:**
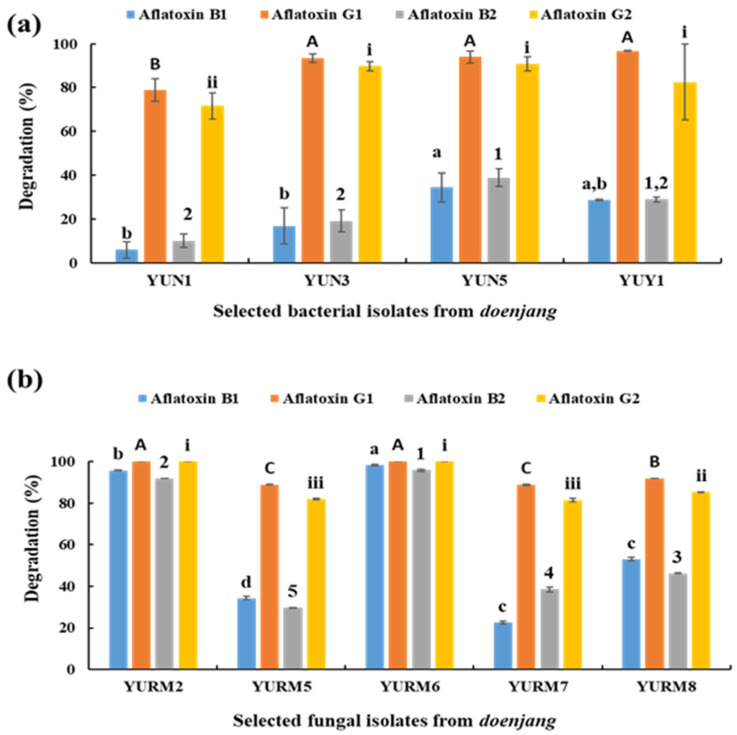
Degradation of aflatoxins by selected bacterial (**a**) and fungal (**b**) isolates from *doenjang*. Each value represents the mean ± standard deviation of three independent experiments. Different lowercase letters (a–d), uppercase letters (A–C), numerical values (1–5), and roman letters (i–iii) represent a significant difference among the levels of aflatoxins B1, G1, B2, and G2 at *p* < 0.05.

Nineteen bacteria were isolated from the *doenjang* samples. Based on microscopic examination and colony characteristics, eight distinct bacterial strains were selected for aflatoxin degradation analysis. The ability to degrade aflatoxins was observed in four isolates, YUN1, YUN3, YUN5, and YUY1, which displayed a 5.93–34.39% degradation capability for aflatoxin B1, 78.97–96.67% for aflatoxin G1, 9.94–38.80% for aflatoxin B2, and 71.62–90.72% for aflatoxin G2 (Figure 6a). These four isolates were selected to examine the degradation of aflatoxins B1 and G1. The results showed that 5.74 ± 3.14%, 19.27 ± 4.24%, 35.97 ± 5.41%, and 27.61 ± 6.09% of aflatoxin B1 and 90.39 ± 2.08%, 86.71 ± 1.47%, 89.74 ± 1.37%, and 84.83 ± 1.36% of aflatoxin G1 were degraded by the bacterial isolates YUN1, YUN3, YUN5, and YUY1, respectively.

Similarly, 14 fungi were isolated from *doenjang* samples. Based on microscopic examination and colony characteristics, nine distinct fungal strains were selected for aflatoxin degradation analysis. The ability to degrade aflatoxins was observed in five isolates, YURM2, YURM5, YURM6, YURM7, and YURM8, which showed a 22.59–98.33% degradation capability for aflatoxin B1, 88.76–100% for aflatoxin G1, 29.57–95.91% for aflatoxin B2, and 81.43–100% for aflatoxin G2 (Figure 6b). Of these five isolates, the three most effective isolates were selected to examine the degradation of aflatoxins B1 and G1. The results showed that 95.81 ± 0.57%, 84.33 ± 4.08%, and 32.71 ± 1.04% of aflatoxin B1 and 100 ± 0.00%, 99.26 ± 0.04%, and 97.29 ± 0.01% of aflatoxin G1 were degraded by fungal isolates YURM2, YURM6, and YURM8, respectively. These results indicated that some fungal species isolated from *doenjang* have a better capacity to degrade aflatoxin B1 compared to the bacterial isolates.

### 3.9. Identification and Phylogenetic Analysis of the Isolated Microorganisms

**Figure 7:**
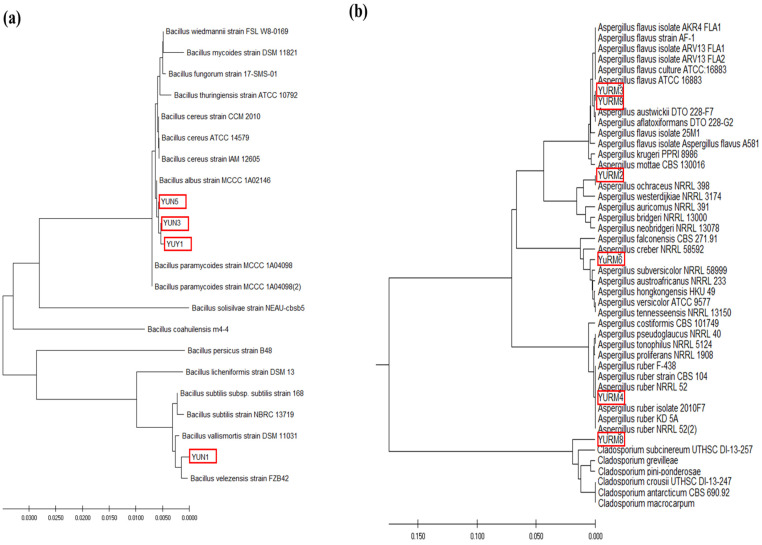
Comparative phylogenetic analysis of aflatoxin-degrading bacteria (**a**) and fungi (**b**) from *doenjang* samples. The phylogenetic tree was constructed using the MEGA6.0 software by employing the neighbor-joining method.

Based on molecular characterization and comparative phylogenetic analysis, the isolated bacterial strains YUN3, YUN5, and YUY1 showed the highest similarity with *Bacillus albus.* The isolated bacterial strain YUN1 was phylogenetically distinct from the other bacterial isolates (YUN3, YUN5, and YUY1) and identified as *Bacillus velezensis* (Figure 7a). The isolated fungi YURM2, YURM6, and YURM8 were found to be phylogenetically distinct and identified as the closest homologs of *Aspergillus ochraceus*, *Aspergillus versicolor*, and *Cladosporium subcinereum*, respectively (Figure 7b). Isolates YURM2 and YURM6 showed 100% sequence similarity with *A. ochraceus* NRRL 398 and *A. versicolor* ATCC9757, respectively, whereas the isolate YURM8 displayed 98% sequence similarity with *C. subcinereum* UTHSC D1-13-257. The isolated *Aspergillus* species (*A. ochraceus* and *A. versicolor*) were found to be non-aflatoxin- and ochratoxin-A-producing fungi and were capable of degrading aflatoxins. These findings were consistent with an earlier report suggesting aflatoxin B1 degradation by atoxigenic *A. flavus* [35]. Similarly, the isolated *A. ochraceus* did not produce ochratoxin A, but was capable of degrading aflatoxin. 

Several studies have previously reported the presence of similar microrganisms such as *Bacillus* sp. [22] and *Aspergillus* sp. [36] in fermented foods that were capable of degrading aflatoxins in different food and feed materials. Similarly, salt-tolerant aflatoxin-degrading microorganisms such as *Candida versatilis* and *Tetragenococcus halophilus* were isolated from soy sauce mash, which is a soybean-based product [37,38]. The presence of similar types of microorganisms in the prepared *doenjang* suggests that they are the major contributors to aflatoxin reduction. It is believed that the identified bacteria (YUN1, YUN3, YUN5, and YUY1) and fungi (YURM2, YURM6, and YURM8) from *doenjnag* are the key elements responsible for the reduction of aflatoxins during the fermentation. The isolated fungi YURM3 and YURM9 capable of producing aflatoxin were identified as the closest homologs of *A. flavus*, while the other aflatoxin-producing isolate YURM4 showed higher similarity with *A. ruber* (Figure 7b). Previously published reports suggested aflatoxin production by *A. ruber* [39]. In this current study, *A. ruber* responsible for aflatoxin B1 production was first detected in *doenjang* samples.

*Doenjang* is made of *meju* and salt and is naturally fermented by diverse microbial communities. In a recent study, four batches of *doenjang* and *meju* prepared from different manufacturers were analyzed for their microbial communities [40]. The microbial communities of *meju* varied from batch to batch. However, in some batches, the microbial communities in *meju* and *doenjang* were similar. Some microorganisms such as *Aspergillus*, *Bacillus,* and *Enterococcus* were present in all *meju* and *doenjang* samples [40], which supports the current findings that most of the isolated bacteria and fungi belong to the genera *Bacillus* and *Aspergillus*, respectively. These results are also supported by previous studies in which *Pediococcus*, *Enterococcus*, and *Bacillus* were reported as dominant bacteria in *doenjang* and *ganjang* [4,41]. Few other studies reported only *Enterococcus* and *Bacillus* as the dominant genera in *meju* and *doenjang* [6,42]. *Aspergillus* is a dominant fungus found in most soybean fermented foods and contributes to flavor and taste [43]. In this study, *Cladosporium subcinereum* was also isolated from *doenjang* and showed aflatoxin degradation ability. This is also supported by previous studies, which reported the isolation of *Cladosporium* sp. from *meju* [44].

### 3.10. Toxicity Determination in Human Skin Fibroblast Cells

**Figure 8:**
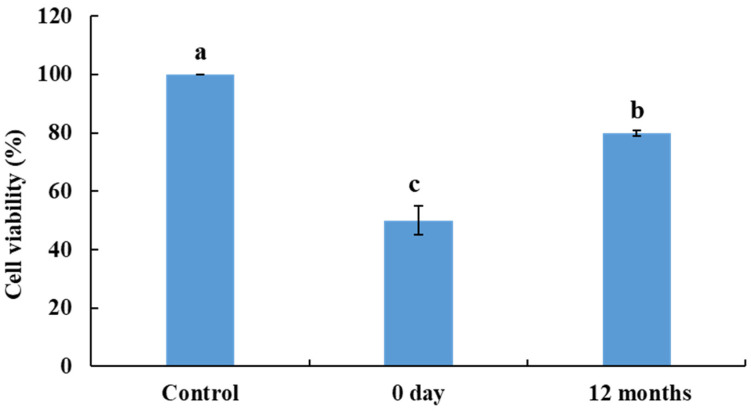
Viability of human skin fibroblasts in the presence of aflatoxin and its degradation products extracted on Day 0 and 12 months of fermentation from *doenjang* spiked with 100 µg/kg each of aflatoxins B1, G1, B2, and G2. Different letters (a–c) represent significant difference at *p* < 0.05.

The toxicity of aflatoxin and its degradation products in *doenjang* was examined using a cell viability assay. Aflatoxin and aflatoxin degradation products were extracted from *doenjang* samples collected on Day 0 and after 12 months of fermentation and examined for their effect on the viability of human skin fibroblasts. The extracted samples from Day 0 exhibited reduced human skin fibroblast cell viability (49.94 ± 4.96%) against the 100% cell viability of the control cells (without any treatment) (Figure 8). In contrast, cells exposed to the samples extracted from 12 months of fermentation displayed a significantly (*p* < 0.05) higher cell viability of 79.93 ± 1.07%, compared to that of the sample extracted on Day 0 (Figure 8). These results indicate that during the fermentation, aflatoxin was degraded by the diverse microorganisms present in *doenjang* into less toxic metabolites, and consequently, the toxicity was reduced. These results are supported by earlier studies suggesting the role of *Pseudomonas putida*, *Bacillus subtilis*, and *Rhodococcus erythropolis* in reducing aflatoxin toxicity towards HeLa cells [45,46,47].

## 4. Conclusions

A time-dependent reduction in aflatoxin levels was observed in *doenjang* samples spiked with aflatoxins, suggesting that native microorganisms are the major contributor involved in the removal of these toxins. Moreover, the high salt concentration of *doenjang,* a unique physiochemical characteristic, prevents the growth of toxin-producing fungi and, thus, aflatoxin production. It was also observed that the aflatoxin contamination in *doenjang* arises mainly during the *meju* preparation stage; therefore, *meju* should be prepared under extremely hygienic conditions using good-quality grains. In conclusion, *doenjang,* which contains high levels of salt, is a complex food with diverse microorganisms that have aflatoxin degradation potential and are associated with the reduction of aflatoxin levels during fermentation. Therefore, storage of *doenjang* in the ambient environment for a prolonged time is advisable for the reduction of aflatoxins below the toxicity limit. Furthermore, the results of the study support the potential utilization of the microorganisms isolated from *doenjang* as starter cultures for the preparation of aflatoxin-free *doenjang* and as biological tools to clean up aflatoxins in various feed and food materials.

## Figures and Tables

**Table 1:** Identification and general characteristics of aflatoxin-producing fungi isolated from *doenjang* and their mycotoxin production ability at different salt concentrations.

Characteristics		Isolate YURM3	Isolate YURM4	Isolate YURM9
**Surface view**		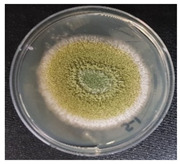	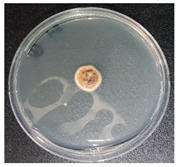	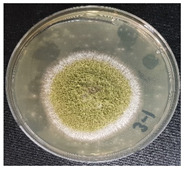
**Microscopic view**		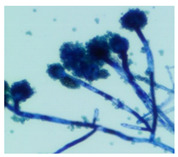	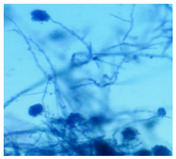	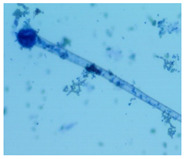
**Identification** **(ITS sequencing)**		*Aspergillus flavus*	*Aspergillus ruber*	*Aspergillus flavus*
**Growth temperature**		28 °C ± 2.0	28 °C ± 2.0	28 °C ± 2.0
**Growth pH**		5.6 ± 0.2	5.6 ± 0.2	5.6 ± 0.2
**Growth at different salt concentrations**	0%	**+++++**	**+++++**	**+++++**
	12%	**+++**	**++**	**+++**
	14%	No growth	No growth	No growth
	16%	No growth	No growth	No growth
	18%	No growth	No growth	No growth
	20%	No growth	No growth	No growth
**Toxin production at different salt concentrations** **(µg/L)**	0%	104.11	53.88	144.54
	12%	79.27	0	88.22
	14%	Not detected	Not detected	Not detected
	16%	Not detected	Not detected	Not detected
	18%	Not detected	Not detected	Not detected
	20%	Not detected	Not detected	Not detected

++ represents low growth, +++ represents moderate growth, +++++ represents high growth.

## Data Availability

Data is contained within the article.

## Supplementary Materials

The following are available online at https://www.mdpi.com/article/10.3390/jof8020190/s1, Figure S1: Standard curve for (a) aflatoxin B1, (b) aflatoxin G1, (c) aflatoxin B2, and (d) aflatoxin G2; Figure S2: Quantification of (a) aflatoxin B1, G1, B2, and G2 and (b) total aflatoxins in *doenjang* samples at 4 °C and *doenjang* samples (autoclaved and un-autoclaved) at room temperature at 12 months of storage; Table S1: Limit of detection (LOD) and limit of quantification (LOQ) for aflatoxin B1, G1, G2, and B2; Table S2: Aflatoxin analysis in non-contaminated *doenjang* during 12 months fermentation.
