# Supplementary material for: Aflatoxin Reduction and Retardation of Aflatoxin Production by Microorganisms in Doenjang during a One-Year Fermentation
Source: J Fungi (Basel). 2022 Feb 15;8(2):190. doi: 10.3390/jof8020190 (PMC8879751; doi:10.3390/jof8020190)
Supplement: Supplementary file 1 [file jof-08-00190-s001.zip › jof-1531759-SI.pdf]

Calibration curve for the quantification of aflatoxins

A calibration curve using the standard aflatoxin B1, G1, B2, and G2 in the concentration range (0.25-20 µg/L) was prepared (Figure S1). The calibration curve was prepared between various concentrations of standard aflatoxins vs the corresponding peak area. For the aflatoxin quantification in doenjang samples, a line of regression was plotted from the calibration curve. Based on used concentrations of aflatoxin B1, G1, B2, and G2 and their corresponding area limit of detection (LOD) and limit of quantification (LOQ) was derived statistically (Table S1).

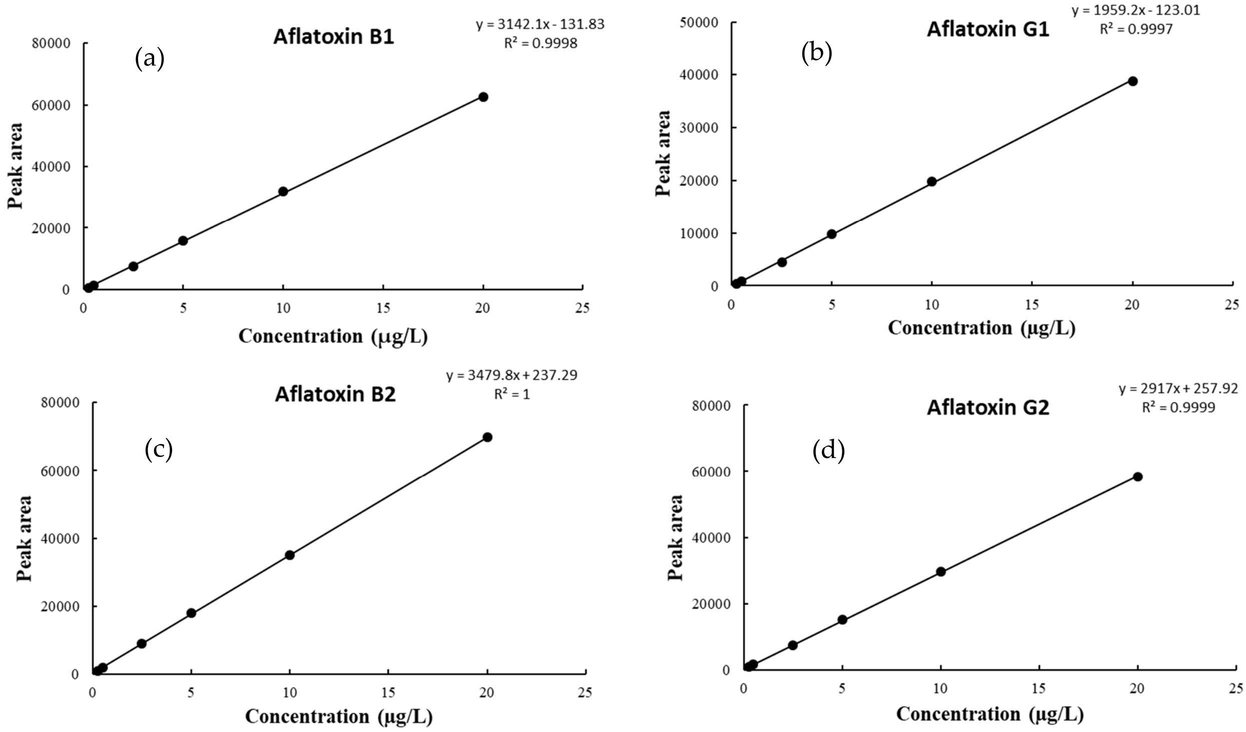

Figure S1: Standard curve for (a) aflatoxin B1, (b) aflatoxin G1, (c) aflatoxin B2, and (d) aflatoxin G2.

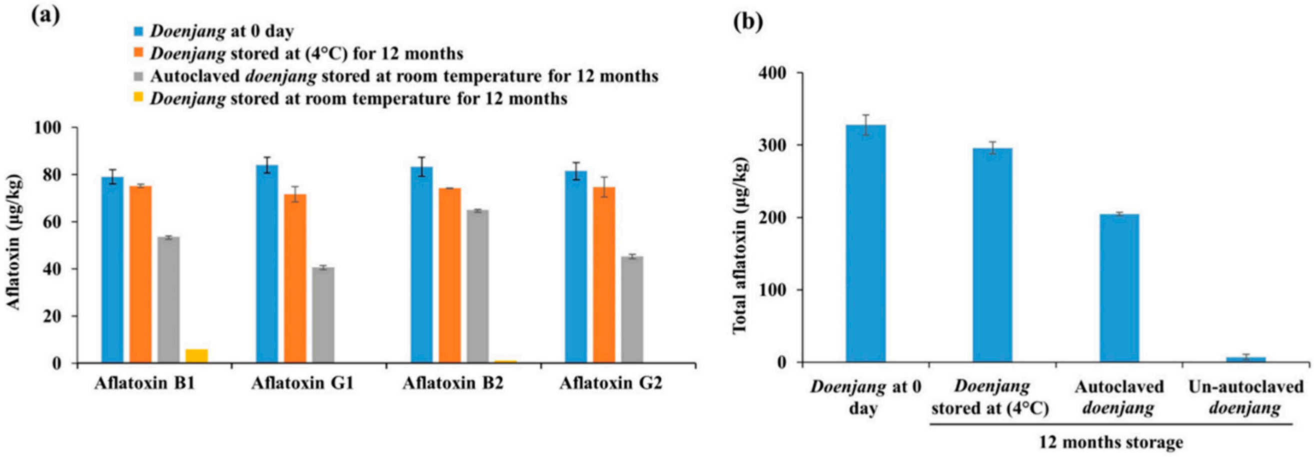

Figure S2: Quantification of (a) aflatoxin B1, G1, B2, and G2 and (b) total aflatoxins in *doenjang* samples at 4°C and *doenjang* samples (autoclaved and un-autoclaved) at room temperature at 12 months of storage.

Table S1: Limit of detecton (LOD) and limit of quantification (LOQ) for aflatoxin B1, G1, G2, and B2

	Standard error intercept	Standard deviation intercept	Slope	R^2	LOD (μg/L)	LOQ (μg/L)
**Aflatoxin B1**	193.96	475.11	3142.1	0.99	0.49	1.51
**Aflatoxin G1**	147.04	360.18	1959.2	0.99	0.60	1.83
**Aflatoxin B2**	84.62	207.29	3479.8	1.00	0.19	0.59
**Aflatoxin G2**	108.05	264.66	2917.0	0.99	0.29	0.90

Table S2: Aflatoxin analysis in non-contaminated *doenjang* during 12 months fermentation

Fermentation (months)	Aflatoxin B1 (μg/kg)	Aflatoxin G1 (μg/kg)	Aflatoxin B2 (μg/kg)	Aflatoxin G2 (μg/kg)
0	0.00 ± 0.00	0.00 ± 0.00	0.00 ± 0.00	0.00 ± 0.00
1	0.00 ± 0.00	0.00 ± 0.00	0.00 ± 0.00	0.00 ± 0.00
2	0.00 ± 0.00	0.00 ± 0.00	0.00 ± 0.00	0.00 ± 0.00
3	0.00 ± 0.00	0.00 ± 0.00	0.31 ± 0.04	0.26 ± 0.10
4	0.00 ± 0.00	0.00 ± 0.00	0.00 ± 0.00	0.00 ± 0.00
5	0.00 ± 0.00	0.00 ± 0.00	0.00 ± 0.00	0.00 ± 0.00
6	0.00 ± 0.00	0.00 ± 0.00	0.00 ± 0.00	0.00 ± 0.00
8	0.00 ± 0.00	0.00 ± 0.00	0.00 ± 0.00	0.00 ± 0.00
10	0.00 ± 0.00	0.00 ± 0.00	0.00 ± 0.00	0.00 ± 0.00
12	0.00 ± 0.00	0.00 ± 0.00	0.00 ± 0.00	0.00 ± 0.00
